# Glioma Surgical Aspirate: A Viable Source of Tumor Tissue for Experimental Research

**DOI:** 10.3390/cancers5020357

**Published:** 2013-04-03

**Authors:** Bryan W. Day, Brett W. Stringer, John Wilson, Rosalind L. Jeffree, Paul R. Jamieson, Kathleen S. Ensbey, Zara C. Bruce, Po Inglis, Suzanne Allan, Craig Winter, Gert Tollesson, Scott Campbell, Peter Lucas, Wendy Findlay, David Kadrian, David Johnson, Thomas Robertson, Terrance G. Johns, Perry F. Bartlett, Geoffrey W. Osborne, Andrew W. Boyd

**Affiliations:** 1 Brain Cancer Research Unit & Leukaemia Foundation Research Unit, Queensland Institute of Medical Research, Brisbane, QLD 4006, Australia; E-Mails: brett.stringer@qimr.edu.au (B.W.S.); paul.jamieson@qimr.edu.au (P.R.J.); kathleen.ensbey@qimr.edu.au (K.S.E.); zara.bruce@qimr.edu.au (Z.C.B.); andrew.boyd@qimr.edu.au (A.W.B.); 2 Queensland Brain Institute, University of Queensland, Brisbane, QLD 4067, Australia; E-Mails: John_Wilson1@health.qld.gov.au (J.W.); p.bartlett@uq.edu.au (P.F.B.); g.osborne@uq.edu.au (G.W.O.); 3 Kenneth G. Jamieson Department of Neurosurgery, Royal Brisbane and Women’s Hospital, Brisbane, QLD 4024, Australia; E-Mails: Lindy_Jeffree@health.qld.gov.au (R.L.J.); craig_winter@health.qld.gov.au (C.W.); gert_tollesson@health.qld.gov.au (G.T.); scott_campbell@health.qld.gov.au (S.C.); peter_lucas@health.qld.gov.au (P.L.); wendy_findlay@health.qld.gov.au (W.F.); david_kadrian@health.qld.gov.au (D.K.); david_johnson@health.qld.gov.au (D.J.); 4 Cancer Services, Royal Brisbane and Women’s Hospital, Brisbane, QLD 4024, Australia; E-Mails: Po-Ling_Inglis@health.qld.gov.au (P.I.); suzanne_allan@health.qld.gov.au (S.A.); 5 Pathology Department, Royal Brisbane and Women’s Hospital, Brisbane, QLD 4024, Australia; E-Mail: Thomas_Robertson@health.qld.gov.au; 6 Oncogenic Signalling Laboratory, Centre for Cancer Research, Monash Institute of Medical Research, Melbourne, VIC 3168, Australia; E-Mail: terry.johns@monash.edu; 7 Department of Medicine, University of Queensland, Brisbane, QLD 4006, Australia

**Keywords:** glioblastoma, glioma, brain cancer, CUSA, surgical aspirate, multiplex flow cytometric analysis, brain cancer stem cells, tumor heterogeneity

## Abstract

Brain cancer research has been hampered by a paucity of viable clinical tissue of sufficient quality and quantity for experimental research. This has driven researchers to rely heavily on long term cultured cells which no longer represent the cancers from which they were derived. Resection of brain tumors, particularly at the interface between normal and tumorigenic tissue, can be carried out using an ultrasonic surgical aspirator (CUSA) that deposits liquid (blood and irrigation fluid) and resected tissue into a sterile bottle for disposal. To determine the utility of CUSA-derived glioma tissue for experimental research, we collected 48 CUSA specimen bottles from glioma patients and analyzed both the solid tissue fragments and dissociated tumor cells suspended in the liquid waste fraction. We investigated if these fractions would be useful for analyzing tumor heterogeneity, using IHC and multi-parameter flow cytometry; we also assessed culture generation and orthotopic xenograft potential. Both cell sources proved to be an abundant, highly viable source of live tumor cells for cytometric analysis, animal studies and *in-vitro* studies. Our findings demonstrate that CUSA tissue represents an abundant viable source to conduct experimental research and to carry out diagnostic analyses by flow cytometry or other molecular diagnostic procedures.

## 1. Introduction

High-grade glioma (HGG) is the most common malignant primary brain cancer. Standard treatment involves surgical resection followed by post-operative radiation and adjuvant temozolomide chemotherapy [1]. Therapy is almost never curative; even with the current standard of care, the median survival is less than 15 months and only about 10% of patients survive two years without disease recurrence [2]. The molecular pathology of HGG is complex and diverse, reflecting a highly heterogeneous, treatment refractory tumor with a very poor prognosis. There is a pressing need to further understand the heterogeneity of HGG and to identify resistant subpopulations. A better understanding of the biology of these sub-populations could lead to novel and effective molecularly-targeted therapeutic strategies. This in turn requires the availability of viable tumor tissue for molecular analysis.

Glioma surgery is often challenging due to the soft, friable texture of the tumor and the need to preserve adjacent cerebral tissue. The Cavitational Ultrasonic Surgical Aspirator (CUSA) allows for gentle, precise removal of tumor, and can be used to define and resect tumour margins. This device allows for removal of tumor in a small field by fragmenting tissue whilst irrigating and aspirating continuously into a sterile waste bottle. As the diagnosis of HGG must be made based on histopathology, and the tumors are often heterogeneous, the majority of tissue that has not been fragmented must be made available for clinical pathology. This limits the availability of tissue for research purposes. 

In order to improve research accessibility to patient samples, we investigated the use of cells harvested using the CUSA. Previous studies have made use of tumor obtained using the CUSA: the tissue fragments were used for diagnostic procedures [3], cell culture [4,5,6] and for detection of oncogene amplifications [7]. This work extends these findings and validates the use of tumor tissue derived from the CUSA as an abundant and viable source of cells for analysing HGG heterogeneity using multi-parameter flow cytometry. Tumor cells sorted from both solid and liquid fractions can readily be cultured using specialised conditions and were capable of initiating intracranial tumor xenografts *in vivo.* In addition CUSA-derived tissue has proven effective for isolation of DNA/RNA, and for performing immunohistochemistry (IHC). This technique represents an approach for investigators to gain further insight into the heterogeneity and underlying biology of HGG. 

## 2. Results and Discussion

### 2.1. Analysis of CUSA-Derived Tissue

**Figure 1:**
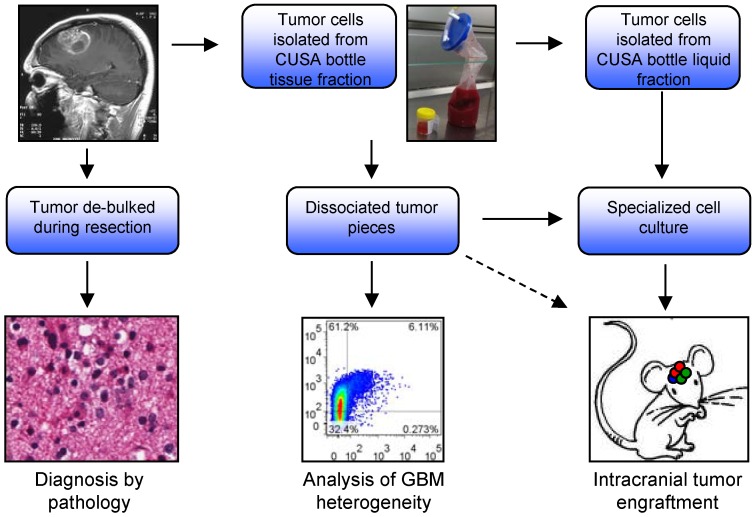
Diagram of experimental processes performed on surgical aspirate. Tumor margins can be resected using a cavitational ultrasonic surgical aspirator (CUSA) which deposits liquid (blood and irrigation fluid) and tissue waste into a sterile bottle for disposal. We isolated these cells and analyzed three aspects: culture generation, xenograft potential and analysis of marker expression using multiplex flow cytometry.

**Figure 2:**
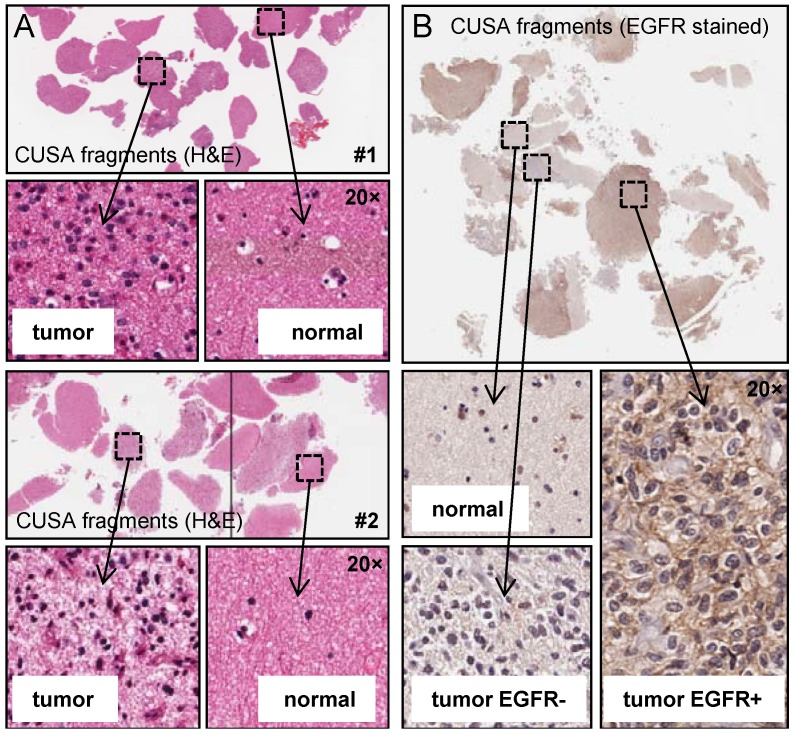
Intratumoral heterogeneity can be detected by IHC in CUSA surgical aspirate. (**a**) H&E sections were prepared from HGG specimens resected using the CUSA, both tumor and normal tissue were identified histologically (data for two specimens are shown); (**b**) IHC staining for a HGG-specific marker (EGFR) of CUSA tissue fragments showing intratumoral heterogeneity, tumor sections stained both positive and negative for EGFR, while normal tissue was negative.

Our primary aim was to investigate the use of tissue obtained from the CUSA aspirated material to isolate tumor cells for research. The CUSA aspirate is typically discarded making this a potential untapped resource of valuable tumor material. We isolated tumor cells from two fractions within the aspirate bottle: One, cells derived from solid tissue fragments (tissue isolated) and two, single cells suspended in the aspirated blood and irrigation fluid (liquid isolated). We assessed these populations for generating tumor cell cultures, and orthotopic xenograft potential. Fresh tissue isolated tumor was also analyzed for tumor heterogeneity using multi-parameter flow cytometry (Figure 1). Firstly, we analyzed CUSA-derived tissue fragments histologically using H&E staining (Figure 2A). Results show fragments of viable tumor tissue are readily detectable. Whilst the majority of fragments contained tumor cells, there were also fragments of what appear to be normal brain tissue. This was expected given the CUSA device is used to resect the invasive edge of the tumor where it abuts normal brain, and a small margin is often resected if the surrounding brain is not eloquent. Unlike tumor tissue obtained from the central tumor mass, where tumor quality is often poor due to significant necrosis and apoptosis, tissue fragments obtained from the CUSA were highly viable. Recent studies have reported intratumoral heterogeneity showing mutually exclusive expression of several receptor tyrosine kinases including EGFR [8,9,10]. We therefore performed IHC for EGFR to determine if intratumoral heterogeneity could be observed using CUSA tissue fragments (Figure 2B). Staining revealed tumor fragments that were both positive and negative for EGFR, while normal tissue fragments were also negative as expected. These results were very encouraging and show that CUSA tissue fragments represent a viable approach to further investigate intratumoral heterogeneity.

### 2.2. Analysis of CUSA-Isolated Tumor Cells

Our next goal was to produce cultures from both tissue and liquid isolated tumor fractions. To grow the cell fractions we selected two culture conditions; RPMI containing serum (10%) and an adherent, serum-free system, which allows for primary glioma specimens to be grown as an undifferentiated monolayer of cells on a basement membrane of laminin. Termed glioma neural stem (GNS) cultures, primary glioma lines derived in this way exhibit gene expression patterns and differentiation behaviour that correlate with specific neural progenitor subtypes, and also allow the use of more traditional cell-based techniques [11]. In total we collected 48 CUSA aspirate samples. We routinely prepared cells for culture from the tissue isolated fraction by mechanical and enzymatic dissociation. We were able to generate sustainable cell cultures in serum conditions with a moderate success rate of 35% (17/48). GNS culture conditions grown on laminin were markedly better with a success rate of 77% (37/48) (Table A1 summarized in the appendix). This far exceeded our previous attempts using tissue pieces resected from the main tumor mass using standard serum culture conditions. To assess both tissue and liquid isolated tumor fractions we used a Fluorescence Activated Cell Sorting (FACS) approach. 

**Figure 3:**
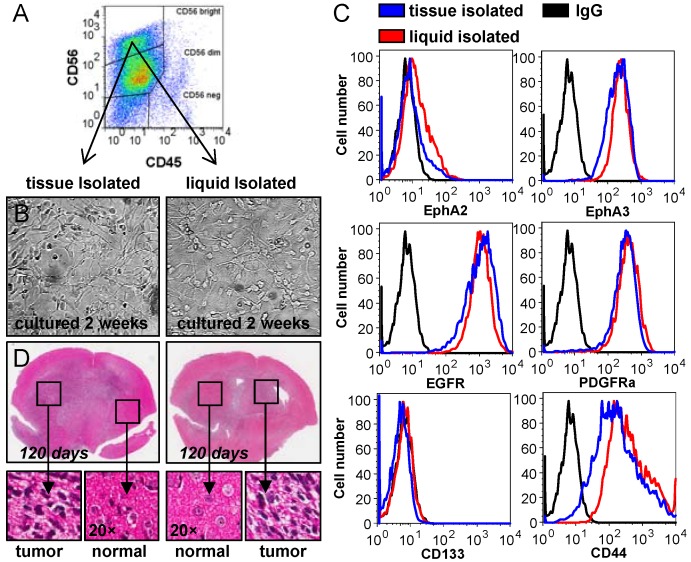
Tumor cells can be reliably isolated and analyzed from CUSA surgical aspirate. (**a**) Brain (normal and tumor) cells were selected by positive staining for neural cellular adhesion molecule (NCAM/CD56) which is expressed on neurons and glia; hematopoietic cells were excluded using CD45; (**b**) CD56 positive cells were isolated by cell sorting from dissociated tissue fragments and from single cells suspended in the liquid fraction of surgical aspirate. Both tissue and liquid isolated cells were grown as GNS and neurosphere cultures and survived for extended passage (>6). Cells isolated from these fractions exhibited identical cell morphology and growth characteristics; (**c**) Flow cytometric expression analysis of known glioma markers in the cultured tissue and liquid fractions revealed similar expression patterns identifying that tumor cells could be cultured from both these fractions; (**d**) The tumorigenic potential of the tissue and liquid isolated cell populations was determined by orthotopic (intracranial) injection. 1 × 10^5^ cells were injected into the right hemisphere of immuno-compromised NOD/SCID mice. H&E sections revealed invasive tumors formed throughout the brain in 120 days using both cultured tissue and liquid isolated tumor fractions.

To select cells of neural origin and to exclude cells of hematopoietic origin, we FACS sorted cells positive for CD56 (NCAM) and negative for CD45 (PTPRC) (Figure 3A). We compared both the tissue and liquid isolated fractions following culture under GNS and neurosphere conditions for two weeks. Growth rate and cell morphology was found to be the same in both fractions (Figure 3B). As heterogeneity is a hallmark of HGG, we also examined the expression of several tumor specific markers found in glioma (Figure 3C). Results showed commensurate marker expression in both fractions using six cell surface markers. This data confirmed that tumor cells could be isolated from not only solid tissue fractions but also as single cells suspended in the liquid fraction. We did note, however, that the number of tumor cells in the liquid fraction varied greatly between samples and often only small numbers of viable cells could be collected. Given that both fractions appeared identical and expressed tumor-specific markers we sought to confirm the tumorigenic potential of these cultures in immune compromised animals. This was assessed by injecting 1 × 10^5^ cells into the right hemisphere of NOD/SCID mice (Figure 3D). In both cases large invasive tumors had formed at 120 days, confirming that tumor initiating cells could indeed be isolated and cultured from both tissue and liquid CUSA material. In addition we selected 10 GNS lines generated from tissue CUSA material and assessed tumorigenic potential in NOD/SCID mice. All 10 lines exhibited strong growth potential *in vitro* and readily formed orthotopic tumors in mice (Table A1 summarized in the appendix).

To determine if tumor fragments could be routinely used to perform expression screens, we analyzed the expression of EphA2 in HGG tumor resected as solid tissue pieces, compared to HGG tumor fragments derived from the CUSA. EphA2 is a member of the Eph receptor tyrosine kinase family. We selected this cell surface receptor as it is known to contribute to glioma tumorigenic potential and is over expressed in glioma but not normal brain [12,13,14].

**Figure 4:**
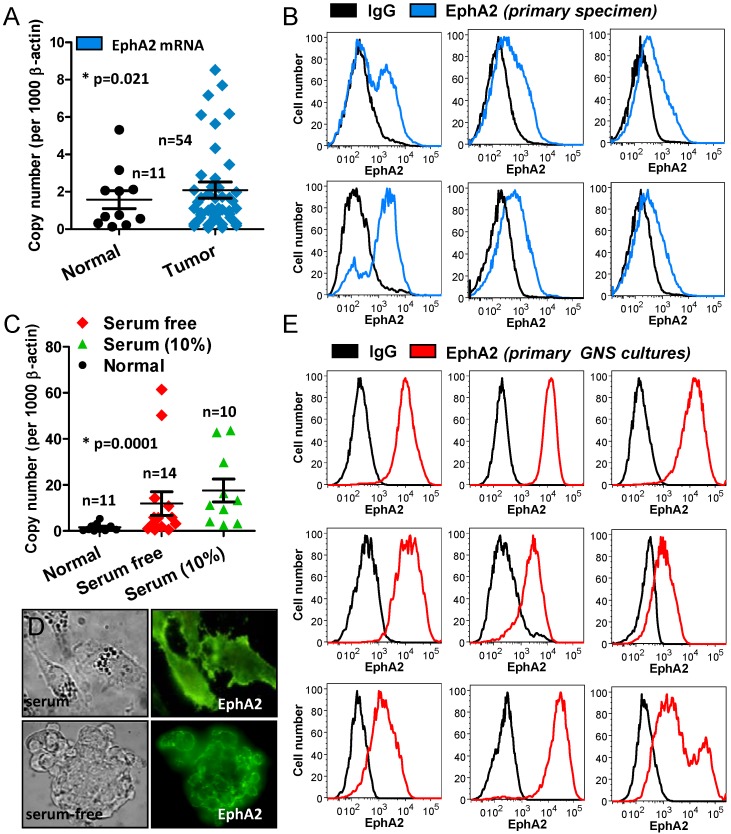
CUSA surgical aspirate can be used to perform tumor expression screens.(**a**) qPCR analysis of EphA2 expression in solid HGG tissue specimens (n = 54) compared to normal brain specimens (n = 11) shows EphA2 is significantly (*p* = 0.021) elevated; (**b**) Flow cytometric analysis of EphA2 expression in CUSA-derived patient specimens (n = 6), shows EphA2 is detectable in these specimens; (**c**) qPCR analysis of EphA2 expression in cultured glioma specimens including both serum-free (n = 14) and serum (n = 10) conditions compared to normal brain specimens (n = 11) shows EphA2 is significantly (*p* = 0.021) expressed in culture; (**d**) Immunofluorescent staining of EphA2 shows strong membrane staining in both serum and serum-free tumorsphere cultures; (**e**) Flow cytometric analysis of EphA2 expression in GNS cultures isolated from CUSA tissue and cultured for >2 weeks (n = 9), shows EphA2 is highly expressed in these cultures.

Firstly we analyzed expression by qPCR in 54 HGG tissue specimens compared to 11 normal brain tissues (Figure 4A). Similar to previously published findings, our data showed EphA2 expression was significantly (*p* = 0.021) elevated above normal brain. Next we dissociated tissue isolated from six CUSA waste bottles and analyzed the fresh tissue by flow cytometry (Figure 4B). In each case EphA2 was readily detectable; these findings show that tissue isolated from the CUSA can be used to undertake tumor expression screens. We also examined the expression of EphA2 by qPCR in 24 cell lines grown in both serum-free, n = 14, and serum containing conditions, n = 10 (Figure 4C). We found that, in general, EphA2 was expressed at higher levels in culture than in the corresponding patient tissues. There was no significant difference between cells cultured in either serum-free or 10% serum media; overall EphA2 was significantly elevated in culture above normal brain (*p* = 0.0001). Immunofluorescence staining of EphA2 in both serum-grown and serum-free tumorspheres showed strong membrane EphA2 staining (Figure 4D). We also examined EphA2 by flow cytometry in 9 primary GNS cultures created from tissue isolated CUSA material (Figure 4E). In each case EphA2 was highly expressed in these cultures. Taken together this data highlights that tumor tissue, isolated using the CUSA, provides a viable, abundant source of cells to reliably generate cell cultures, and to undertake expression screens on primary patient specimens.

### 2.3. Analysis of Tumor Heterogeneity Using Multi-Parameter Flow Cytometry

To explore the potential that CUSA tissue could be used to assess HGG heterogeneity we employed multi-parameter flow cytometry using a panel of cell surface markers. This allowed the examination of multiple parameters on each cell and provides information on the correlated expression levels of selected cell surface receptors at a given point in time. 

**Figure 5:**
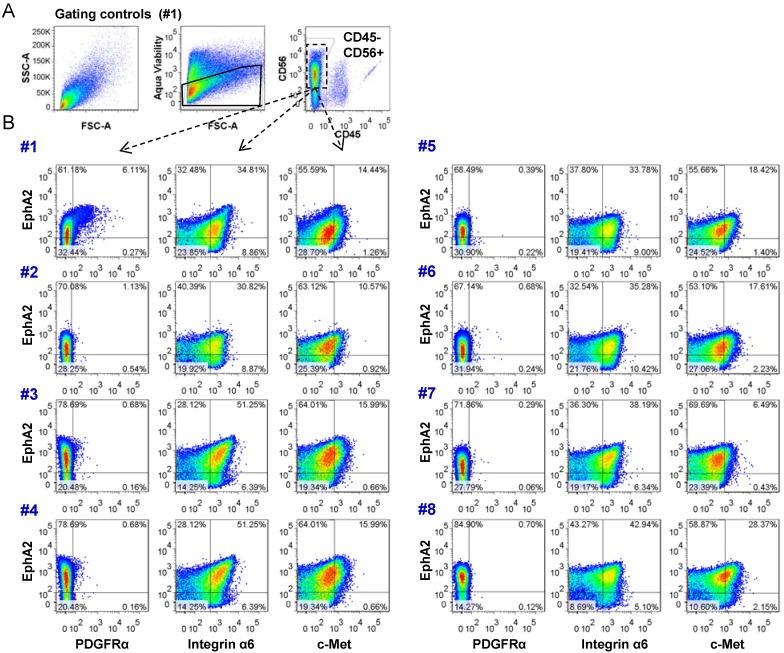
CUSA surgical aspirate can be used to analyze tumor heterogeneity using multiplex flow cytometry. (**a**) Cells staining negative for aqua viability dye were gated and plotted to examine the NCAM/CD56 positive CD45 negative populations; (**b**) Multiplex flow cytometric analysis of fresh CUSA-isolated surgical tissue aspirate collected immediately after surgery and processed and stained as described in materials and methods. Expression results are shown for PDGFRα, integrin α6, and c-Met with respect to EphA2.

Heterogeneity within a tumor may compromise the efficacy of a single therapeutic agent; therefore the demonstration of tumor heterogeneity could be therapeutically useful. By identifying functional differences between subpopulations of a given tumor, therapies might be planned to target the chemosensitivities of these populations. Our panel was designed to detect molecules that might predict efficacy for one or more therapeutic agents. Our analysis strategy was firstly to select viable (aqua viability stain), CD56 positive cells and then to exclude CD45 positive cells (Figure 5A). EphA2 was chosen as recent evidence suggests it may be a marker of tumor propagating cells [12]. This receptor was then compared to PDGFRα [15], CD49f (integrin α6) [16], and c-Met [17] (Figure 5B). Multiplex analysis revealed expression of EphA2 in all eight specimens. PDGFRα was primarily low in the majority of specimens with positive populations detected in three specimens (#1, #3, #4). All specimens showed integrin α6 and c-Met were co-expressed with EphA2. Our results clearly show defined sub-populations are present in these specimens. These data now confirm that multiplex analysis of CUSA-resected tumor is a viable approach to explore the heterogeneity of glioma using freshly isolated tumor tissue.

## 3. Experimental Section

### 3.1. Ethics Statement

Patient tissue specimens were collected under approved Queensland Institute of Medical Research and Royal Brisbane and Women’s Hospital human ethics; all samples were obtained following written consent and de-identified. For a list of specimens collected see Table A1 in the Appendix. All animal studies were performed under approved Queensland Institute of Medical Research animal ethics (Project I.D P1173) and carried out in strict accordance with ethical guidelines and use of laboratory animals to minimise animal suffering.

### 3.2. Patient Specimens

Surgical resection was carried out using a CUSA EXcel™ ulstrasonic aspirator (Valleylab, Boulder, CO, USA) at settings determined by the surgical requirements. Typically surgeons selected CUSA settings just high enough to resect tumours easily: Aspiration 20%, Irrigation 1, Amplitude 40% and Tissue Select ++++. At higher settings, considerable amounts of foam accumulate in the aspiration bottle and there is probably cell lysis. Tissue was stored in an isotonic solution (Ringer’s solution) in the CUSA bottle during the procedure. Tissue was removed from the CUSA bottle and processed typically within three hours following resection. The procedure used to process the tissue is outlined in detail in [11]. Briefly, CUSA tissue fragments were pelleted and red blood cells removed by incubation in RBC lysis buffer. Fragments were washed in PBS and mechanically dissociated using a scalpel (10 min) and enzymatically dissociated using Accutase (Sigma, St. Louis, MO, USA) (10 min). Dissociated cells were passed through a 100 µM disposable sterile cell strainer and washed in PBS prior to use or cryopreservation.

### 3.3. Cell Culture

Glioma cells were cultured using three methods: (1) RPMI supplemented with 10% FBS, (2) as tumorspheres using StemPro^®^ NSC SFM (Invitrogen, Grand Island, NY, USA), (3) as glioma neural stem (GNS) cultures as outlined in detail in [11]. Cells were incubated at 37 °C, 5% CO_2_.

### 3.4. Orthotopic Xenografts

GNS cells were cultured for two weeks and 1 × 10^5^ cells injected, at a depth of 3 mm using a stereotactic frame, into the right hemisphere of 5 week old NOD/SCID mice. Animals were monitored for signs of tumor formation (rough coat, hunching, and weight loss) and euthanized as per ethical practices. 

### 3.5. Relative Quantitation by Real-Time PCR

RNA was extracted using TRIzol^®^ (Invitrogen). First strand cDNA was synthesized using random hexamers and Superscript™ III (Invitrogen). Real-Time PCR was carried out using SYBR^®^ Green PCR Master Mix (Invitrogen). Cycling conditions were 15 min at 95 °C and 30 cycles of 30 s at 95 °C, 30 s at 55 °C, and 30 s at 72 °C. Primers sequences: EphA2 GGGACCTGATGCAGAACATC (sense) AGTTGGTGCGGAGCCAGT (anti-sense) and β-actin CACACTGTGCCCATCTACGA (sense) GTGGTGGTGAAGCTGTAGCC (anti-sense).

### 3.6. Immunofluorescence and IHC

Glioma cultures were stained for EphA2 as follows: Cells were washed twice with 5% FCS in PBS. EphA2 was detected using an in-house mouse monoclonal antibody (1F7, 5 µg/mL) for 20 min at RT. Secondary detection antibody was anti-mouse Alexa 488 (Invitrogen) (1:1,000) for 15 min at RT. Cover slips were mounted on glass slides using ProlongGold anti-fade reagent (Invitrogen).

Immunohistochemical staining was performed on 4 µm formalin-fixed/paraffin embedded (FFPE) sections. Sections were subjected to enzyme retrieval with 0.2% pepsin in 0.1 N HCl. Endogenous peroxidase activity was blocked with 1.0% H_2_O_2_, and non-specific binding blocked with 10% goat serum. Primary antibody (Millipore anti-EGFR, clone LA22, cat# 05-104, Billerica, MA, USA) was applied at 5 µg/mL (diluted in 10% goat serum) for 1 h at RT, followed by detection using the MACH 1 HRP-Polymer Detection system (Biocare Medical, Concord, CA, USA). Color was developed in 3,3'-diaminobenzidine (DAB) with H_2_O_2_ as substrate.

### 3.7. Flow Cytometric Analysis and Sorting

Flow cytometric analysis of both primary tissue and cultured cells was performed as follows. Cells were washed once in cold PBS, centrifuged at 300 g for 5 min, supernatant removed then resuspended in PBS. Cells were aliquotted at 5 × 10^6^/mL. Unconjugated primary antibodies were added at 10 µg/mL and incubated for 15 min on ice. Cells were washed and secondary conjugated antibodies were added at a dilution of 1/100. The previous incubation step and wash steps were repeated. 5 µL of conjugated antibodies were added to 100 µL of cells with the following exceptions, CD56 2 µL/100 uL and CD45 3 µL/100 uL. After incubation and washing cells were resuspended in 20 µL of PBS and analyzed on a BD LSR II flow cytometer. Data analysis was carried on FlowJo software (version 7.6.4). Antibodies are listed in Table A2 in the appendix.

Sorting of primary tissue was performed on a BD Influx cell sorter. Briefly, cells were washed once, in PBS, centrifuged at 300 g for 5 min and resuspended in PBS, stained with anti-human CD56 PE (BD, Franklin Lakes, NJ, USA, cat 557711) and anti-human CD45 V450 (BD cat 642275) at volumes scaled up from 500 × 10^3^ cells in 100 µL. Cells were then washed twice and resuspended in PBS and sorted and cultured at 37 °C, 5% CO_2_. Sort conditions were 11.6 PSI sheath pressure; 120 µm nozzle and drop drive frequency using 27.6 KHz. Sort software used was Spigot 6.1.9 and BD Sortware 1.0.

### 3.8. Statistical Analysis

Student’s t-test determined the probability of difference and a *p*-value <0.05 was considered significant. All statistical tests were two-sided.

## 4. Conclusions

It is now well described that HGG is a highly heterogeneous disease. Culture of tumor cells by any means will always be imperfect and in many cases allow researchers to only examine a proportion or subclone of the original specimen. It is therefore critical that researchers use fresh tumor tissue to gain a true representation of the disease state. Our understanding of the genetic and molecular landscape of glioma has increased tremendously in recent years. Despite these advances, clinical outcomes have improved only modestly [18].

We assessed the potential of tissue isolated from the CUSA as a means to provide tumor to conduct studies into glioma heterogeneity and evaluate known HGG markers. We were at first sceptical as to the utility of this tissue, but to our surprise found a viable source of primary tumor sufficient in quantity and quality to meet the demands of both experimental research and evolving molecular diagnostic techniques. There are a number of reasons why tissue resected in this manner may be more viable than conventional solid biopsy specimens. Often the CUSA is used to resect along the interface between tumour and normal tissue. This may contain a higher proportion of migrating and proliferating cells than the central, often necrotic, tumor mass. The cells are then suspended in a physiological electrolyte solution which may maintain homeostasis and protect from oxidative damage. 

The quantity and quality of tissue enabled us, in many cases, to cryopreserve multiple aliquots of fresh dissociated CUSA tissue. This has become a vital resource in our laboratory and has enabled us to return to the original specimen for further analyses. In our hands the frozen tumor cells thaw with high viability and can be used to generate cultures in both GNS and neurosphere conditions, conduct flow cytometric analysis or successfully transfer the patient’s cells to an orthotopic xenograft setting. CUSA tissue also provides quality RNA and DNA and can be used to perform IHC. The only potential drawback of using CUSA isolated tumor cells is the concern of contaminating normal brain. CUSA fragmented tissue contained both tumor and normal, though tumor was often more prevalent. We have now shown that cultures (n = 16) generated from CUSA tissue are tumorigenic in immune compromised animals. Furthermore tumor cells in culture will always proliferate significantly more than normal cells and as such outcompete normal cell populations. Flow cytometric strategies are not significantly compromised by admixing of normal cells as there are now many robust glioma-specific cell surface markers that can be used to effectively exclude any contaminating normal tissue.

Of particular interest was our finding that tumor cells could be analyzed and isolated from the liquid CUSA fraction. Whilst these cells were not always present in great numbers, it did provide an opportunity to directly assess tumor surface marker expression without subsequent tissue preparations. This work paves the way for real time flow cytometric analysis of a patient’s tumor in the operating theatre utilizing rapid-binding antibodies. In principle the relevance of this approach in brain tumor surgery has been demonstrated [19]. Our findings only strengthen the validity of a rapid feedback mechanism which may aid in a treatment regime tailored to the characteristics of a particular tumor. Moreover, this may be useful to investigate circulating HGG cells, a burgeoning field in brain cancer research. A multi-color flow cytometric assay could eventually provide a platform for not only diagnosis, but as an approach to pinpoint therapeutic targets and guide treatment strategies.

## Appendix

**Table A1:** CUSA specimen analysis.

Sample	Tumor Type	Tumor Location	Age	Sex		Serum Culture	GNS Culture	Tumorigenic in Mice
**1**	Normal	Temporal lobe/neocortex	37	Male			√	
**2**	Astrocytoma WHO grade III	Posterior Temporal	38	Male			√	
**3**	Anaplastic ODG WHO grade III	Right frontal	39	Male				
**4**	Diffuse astrocytoma	Left parietal occipital	19	Male		√	√	
**5**	Diffuse astrocytoma	Left temporal	38	Male				
**6**	GBM	Right frontal	26	Male			√	
**7**	GBM	Left frontal parietal	32	Male		√	√	
**8**	GBM	Left frontal	34	Male		√		
**9**	GBM	Left frontal parietal	37	Male			√	
**10**	GBM	Right parietal	37	Female			√	√
**11**	GBM	Right parietal	47	Male		√	√	√
**12**	GBM	Left occipital	51	Male				
**13**	GBM	Left frontal parietal	51	Male		√	√	√
**14**	GBM	Right temporal parietal	51	Male			√	
**15**	GBM	Right frontal parietal	54	Female		√	√	√
**16**	GBM	Left temporal	56	Male		√	√	√
**17**	GBM	Right temporal	57	Male		√	√	
**18**	GBM	Left temporal	57	Female			√	√
**19**	GBM	Right parietal	58	Male		√		
**20**	GBM	Left temporal	58	Female		√	√	
**21**	GBM	Left frontal	58	Female				
**22**	GBM	Left frontal	59	Male			√	
**23**	GBM	Right temporal	59	Male			√	
**24**	GBM	Right temporal	61	Female		√	√	
**25**	GBM	Posterior temporal	61	Female			√	
**26**	GBM	Right parietal	63	Male		√	√	
**27**	GBM	Left temporal	64	Female		√		
**28**	GBM	Left frontal	65	Male			√	
**29**	GBM	-	65	Male			√	
**30**	GBM	Right temporal	58		Female	√		
**31**	GBM	Right temporal	68		Male		√	
**32**	GBM	Right intrinsic	69		Male	√	√	
**33**	GBM	Left temporal	69		Male			
**34**	GBM	Right frontal	74		Male		√	
**35**	GBM	Parietal	74		Female			
**36**	GBM	Left parietal	74		Male		√	√
**37**	GBM	Right frontal	75		Male	√	√	√
**38**	GBM	Right frontal	75		Female		√	
**39**	GBM	Left frontal	84		Female		√	√
**40**	Oligoastrocytoma WHO grade II	Left fronto-temporal	46		Male		√	
**41**	ODG	Right occipital	35		Female		√	
**42**	ODG	Left frontal	35		Male			
**43**	ODG	Right frontal	58		Female		√	
**44**	Recurrent diffuse glioma WHO grade II	Right frontal parietal	40		Female		√	
**45**	Recurrent GBM	Right parietal	48		Male		√	
**46**	Recurrent GBM	Left parietal	52		Male		√	√
**47**	Recurrent GBM	Right parietal	62		Male		√	
**48**	Recurrent GBM	Right parietal	62		Male		√	

GBM: Glioblastoma Multiforme; ODG: Oligodendroglioma; WHO: World Health Organization.

**Table A2:** List of antibodies used in multiplex color staining.

Name	Antibody Clone	Origin	Catalogue Number	Fluorochrome
**CD56**	B159	BD	557747	PE-Cy7
**CD45**	2D1	BD	560178	APC-H7
**Aqua Viability Dye**	-	Molecular Probes	L34957	Aqua
**EphA2**	1F7	In-house	N/A	Unconjugated
**CD140a (PDGFRα)**	aR1	BD	556002	PE
**CD49f (Integrin-α6)**	EBioGoH3	Ebioscience	46-0495-82	PerCp-eFluor710
**c-Met**	LMH85	In-house	N/A	FITC
